# Editorial: Allergen-specific antibodies: from basic science to clinical application

**DOI:** 10.3389/falgy.2025.1568735

**Published:** 2025-02-26

**Authors:** Sabine Flicker, Mats Ohlin, Amanda Atanasio, Anna Pomés

**Affiliations:** ^1^ Institute of Pathophysiology and Allergy Research, Center for Pathophysiology, Infectiology and Immunology, Medical University of Vienna, Vienna, Austria; ^2^ Department of Immunotechnology and SciLifeLab, Lund University, Lund, Sweden; ^3^ Immunology and Inflammation, Regeneron Pharmaceuticals, Inc., Tarrytown, NY, United States; ^4^ Basic Research, InBio, Charlottesville, VA, United States

**Keywords:** monoclonal antibody, antibody epitopes, IgE, IgG, IgA, antibody isotype, allergy, antibody-based approaches for diagnosis and immunotherapy

**Editorial on the Research Topic**
Allergen-specific antibodies: from basic science to clinical application

IgE antibodies are key players in the initiation, progession and maintenance of type I hypersensitivity, a common public health problem affecting about one third of the world´s population. Advanced technologies initially exploiting phage display combinatorial libraries (1–3), and more recently human hybridomas (4, 5) and single B cell sequencing (6) have established the basis to generate human IgE monoclonal antibodies (hIgE mAbs) that are facilitating their thorough investigation. These efforts expanded the original studies that identified conformational IgE epitopes on Bos d 5 and Phl p 2 using combinatorial libraries (7, 8) and led to define for the first time human IgE epitopes using hIgE mAbs with the pairing of the heavy and light chains as it occurs *in vivo* (4, 9, 10). The acquired knowledge is a vital prerequisite to understand underlying mechanisms of IgE-mediated allergic disease and to further develop strategies to prevent IgE-triggered effects and hence allergic symptoms. Polyclonal but also monoclonal allergen-specific IgG antibodies have been already described to inhibit IgE elicited mode of action primarily by binding allergen and preventing engagement with IgE on the surface of allergic effector cells. Prophylactic administration of allergen-specifc IgG mAbs has been demonstrated to prevent allergic symptoms in birch and cat allergic individuals in early clinical studies, thus providing a foundation for future development and application in the field of allergy (11–14).

The present Special Research Topic “*Allergen-specific Antibodies: From Basic Science to Clinical Application*” offers a current snapshot about the latest findings concerning antibody-based approaches to study the development of allergic disorders, to establish efficient standardisation of allergen extracts and to employ antibodies for allergy treatment. This collection includes three review articles covering antibody isotype diversity in the context of allergy (Platts-Mills et al.), the importance of microbial influence during pregnancy and first year of life (Pirker and Vogl), and the significance of IgE epitope recognition for both developing blocking antibodies and peptide-based immunotherapies (Fernandes et al.). Furthermore, this edition emcompasses one original article describing the establishment of a highly efficient ELISA using human IgG_4_ antibodies to standardize commercially available allergen extracts used for diagnosis and therapy of type I allergy in the US (Rabin et al.), and a narrative mini review article summarizing recent news on the sucessful treatment with omalizumab to manage various food allergies (Ghouri et al.).

Over the last 40 years, two developments have been important in the allergy field: (1) the improvement of techniques for measuring isotype specific antibodies to allergens, and (2) the identification of allergic diseases or phenomena involving isotype-diversity of antibodies. The manuscript by Dr. Platts-Mills et al. highlights the importance of assessing allergen specific antibodies of diverse isotypes, not only IgE, for the investigation of allergic diseases. The review shows the relevance of antibody isotypes in certain allergic diseases with a historical perspective. Compelling evidence is presented for the role of IgG4 in the development of eosinophilic esophagitis and tolerance to cat, and for the presence of IgA and IgG, in addition to IgE, in nasal secretions of pollen allergic patients. On the other hand, IgE recognition of the oligosaccharide galactose-α-1,3-galactose is essential for the alpha-gal syndrome, primarily induced by tick bites, but the role of IgG isotypes on the inflammatory response to the sugar needs further investigation.

Microbial colonization of human mucosal surfaces is an essential component of host immune system development. It's widely recognized that a critical window of time extending from pregnancy through the first year of life exists in which early microbial encounters influence and shape the immune response. However, mechanisms underlying the impact of such interactions remain incompletely understood. Development of asthma and allergic diseases has been associated with microbial dysbiosis, and recent research suggests that both pre- and postnatal antibody production plays a role in shaping the microbiome. In their review, Pirker and Vogl discuss early life factors influencing the composition of the gut microbiome, including delivery and feeding modes, medication and maternal-, geographical- and social factors. They provide a comprehensive review of recent work aimed to better understand innate and adaptive immune mechanisms underlying the microbiota-immune axis and how it may relate to the onset of allergic disease in early life.

The diversity and complexity of B and T cell epitope recognition profiles of adaptive immune responses in relation to allergic disease is an underexplored domain of allergy research and their impact on disease, diagnosis and immunological intervention is poorly understood. In their review, Fernandes et al. thoroughly outline our understanding of epitopes of major Hymenoptera (bee, wasp, and ant) venom allergens. Such proteins display very substantial potential to cause severe, life-threatening allergic disease in sensitized individuals. They furthermore exemplify epitope-based strategies that have been explored for treatment of allergic disease through passive and active immunotherapy in other allergies, and advocate for the importance of an epitope-based strategy in the development of diagnostic and therapeutic approaches for the treatment and prevention of Hymenoptera venom allergies.

Between 10% and 20% of the population suffer from IgE-mediated cat allergy. Cat allergen extracts standardized for the major cat allergen, Fel d 1, are used to diagnose cat allergy but also applied as immunotherapy to prevent allergic symptoms. So far, a radial immunodiffusion assay and polyclonal serum have been utilized to determine the concentration of Fel d 1 in commercially available cat dander and pelt extracts in the US. However, this technology comes along with some limitations including difficulties with interpreting precipitation results and insufficient homogeneity of polyclonal serum. Within their original article Rabin et al. describe the generation and evaluation of human Fel d 1-specific IgE-derived IgG_4_. These antibodies guarantee IgE epitope binding clinically relevant for allergen recognition and the elaboration of a highly reproducible and precise assay based on a selected pair of these human antibodies binding to non-overlapping epitopes. Emphasizing that measurement of Fel d 1 levels is sufficient to detect the potency of cat allergen extracts, they prove their quantitative two-site assay to be equivalent to the radial immunodiffusion currently applied for standardization in the US.

Food allergy is a serious threat implying considerable restriction in daily life for those who are impacted. Injections with omalizumab have been approved by the FDA for the treatment of food allergic adults and children aged one year or older. The narrative mini review written by Ghouri et al. provides a current overview on the efficacy, safety and clinical implications of omalizumab in most frequent food allergies including peanut, milk, shellfish but also fruits. While their summary clearly demonstrated that administration of omalizumab is a major progress in the management of food allergies, it also revealed that the high cost of monoclonal antibodies and insurance coverage constraints represent a huge challenge that needs to be overcome before offering such treatment to affected patients.

In summary, this research topic delivers novel insights into the role of allergen-specific IgE, IgG, IgA and anti-IgE antibodies in the perpetuation of allergy but also informs about potential strategies for more effective diagnosis and treatment of IgE-mediated allergies.
